# Silencing of hsa_circ_0009035 Suppresses Cervical Cancer Progression and Enhances Radiosensitivity through MicroRNA 889-3p-Dependent Regulation of HOXB7

**DOI:** 10.1128/MCB.00631-20

**Published:** 2021-05-21

**Authors:** Xia Zhao, Weilei Dong, Guifang Luo, Jing Xie, Jie Liu, Furong Yu

**Affiliations:** a Department of Gynaecology and Obstetrics, The First Affiliated Hospital of University of South China, Hengyang City, Hunan Province, China

**Keywords:** cervical cancer, hsa_circ_0009035, miR-889-3p, HOXB7

## Abstract

Circular RNAs (circRNAs), a novel type of endogenous noncoding RNAs, have been identified as critical regulators in human carcinogenesis. Here, we investigated the precise actions of hsa_circ_0009035 in the progression and radioresistance of cervical cancer (CC). The levels of hsa_circ_0009035, microRNA 889-3p (miR-889-3p), and homeobox B7 (HOXB7) were detected by quantitative real-time PCR (qRT-PCR) or Western blotting. RNase R and actinomycin D assays were used to assess the stability of hsa_circ_0009035. Cell proliferation, cell cycle progression, apoptosis, migration, and invasion were gauged with Cell Counting Kit-8 (CCK-8), flow cytometry, and transwell assays. Cell colony formation and survival were determined by the colony formation assay. Targeted correlations among hsa_circ_0009035, miR-889-3p, and HOXB7 were examined by the dual-luciferase reporter, RNA immunoprecipitation (RIP), or RNA pulldown assay. Animal studies were performed to evaluate the impact of hsa_circ_0009035 on tumor growth. We found that hsa_circ_0009035 was highly expressed in CC tissues and cells, and it was associated with the radioresistance of CC patients. Moreover, the silencing of hsa_circ_0009035 inhibited CC cell proliferation, migration, invasion, and it enhanced apoptosis and radiosensitivity *in vitro* and weakened tumor growth *in vivo*. Mechanistically, hsa_circ_0009035 directly targeted miR-889-3p by binding to miR-889-3p, and hsa_circ_0009035 modulated HOXB7 expression through miR-889-3p. HOXB7 was a functional target of miR-889-3p in regulating CC progression and radioresistance *in vitro*, and hsa_circ_0009035 modulated CC progression and radioresistance *in vitro* by miR-889-3p. Our current study first identified hsa_circ_0009035 as an important regulator of CC progression and radioresistance at least in part through targeting the miR-889-3p/HOXB7 axis, highlighting its significance as a potential therapeutic target for CC treatment.

## INTRODUCTION

Cervical cancer (CC) ranks fourth in both incidence and mortality among women globally. Approximately 570,000 new cases were diagnosed and ∼311,000 deaths occurred in 2018 (1). Although high-quality screening programs have significantly decreased the incidence and mortality rates over the last few decades, the clinical outcome of patients with advanced-stage disease is still very poor (2, 3). Crucial modulators of CC tumorigenesis, including noncoding RNAs (ncRNAs), are under investigation (4–6). A clearer understanding of the precise components of these regulatory molecules would provide a unique opportunity to design therapeutics with molecular targets.

As a novel type of ncRNAs, circular RNAs (circRNAs) have been shown to be critical regulators in cancer pathogenesis (7). Furthermore, recent reports demonstrate that some circRNAs can function as either tumor drivers or antitumor players in CC by working as microRNA (miRNA) inhibitors (8, 9). For example, Cai et al. revealed that hsa_circ_0000263 contributed to CC tumorigenesis by directly targeting miRNA 150-5p (miR-150-5p) (10). Song et al. highlighted the oncogenic property of hsa_circ_101996 in CC by reducing miR-8075 activity (11). Guo and colleagues identified the important involvement of hsa_circ_0023404 in CC carcinogenesis and chemoresistance via the regulation of miR-5047 (12). Interestingly, hsa_circ_0009035, engendered by the backsplicing of exons of Rac GTPase-activating protein 1 (RACGAP1), was found as one of the top four upregulated circRNAs in radioresistant HeLa cells (13). Nonetheless, the precise, critical actions of hsa_circ_0009035 in CC progression and radioresistance remain underexplored.

Recently, aberrant up- and downregulation of miRNAs in CC carcinogenesis has been widely reported (14). Sun and colleagues identified that miR-889-3p (also called miR-889), a striking underexpressed miRNA in CC, functioned as a tumor suppressor in CC through targeting fibroblast growth factor receptor 2 (15). When we used computer algorithms to search a novel circRNA/miRNA/mRNA regulatory network mediated by hsa_circ_0009035, we found two targeted relationships among hsa_circ_0009035, miR-889-3p, and homeobox B7 (HOXB7), a critical player in CC development (16). For these reasons, we set out to identify how hsa_circ_0009035 regulated CC progression and radioresistance and whether the hsa_circ_0009035/miR-889-3p/HOXB7 axis was an indeed functionally regulatory network.

Here, we provided evidence that hsa_circ_0009035 regulated CC progression and radiosensitivity. Furthermore, we identified the miR-889-3p/HOXB7 regulatory network as a novel molecular mechanism for the regulation of hsa_circ_0009035.

## RESULTS

### hsa_circ_0009035 was highly expressed in CC tissues and cells.

**FIG 1:**
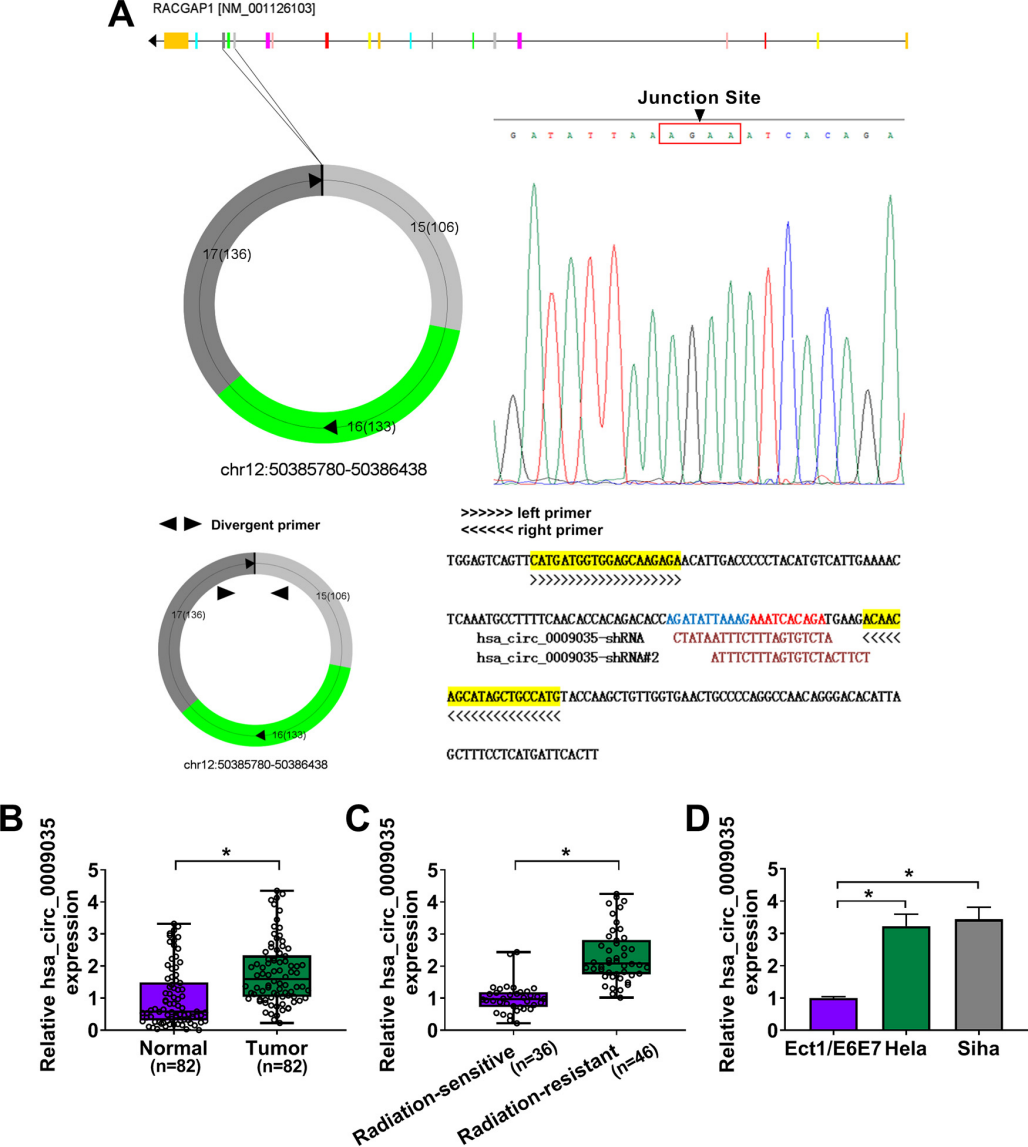
hsa_circ_0009035 expression was upregulated in CC tissues and cells. (A) Schematic diagram of hsa_circ_0009035 formation via the backsplicing of exons 15, 16, and 17 of RACGAP1 mRNA and identification of the backsplice junction sequences by Sanger sequencing. The design details of the qRT-PCR primer (divergent primer) and shRNAs of hsa_circ_0009035 are also shown. (B to D) Relative hsa_circ_0009035 expression by qRT-PCR in 82 pairs of CC tissues and adjacent normal tissues (B), CC tissues from 36 primary patients (defined as radiation-sensitive CC) and 46 recurrent patients after radiation treatment (defined as radiation-resistant CC) (C), and Ect1/E6E7, HeLa, and Siha cells (D). *, *P < *0.05.

**FIG 2:**
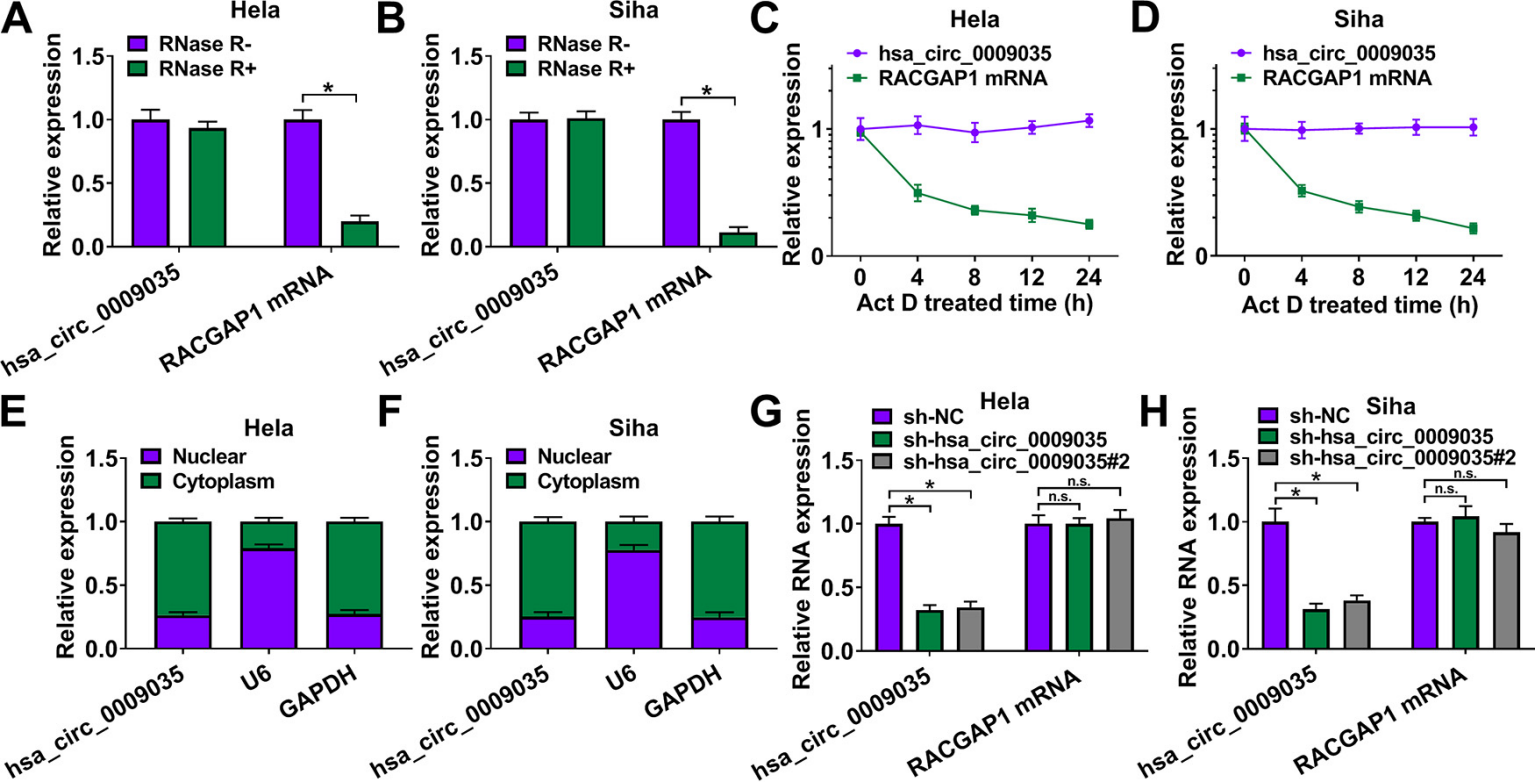
Characterization of hsa_circ_0009035. (A and B) RNase R assays in HeLa and Siha cells. (C and D) Actinomycin D assays in HeLa and Siha cells. (E and F) Subcellular localization assays in HeLa and Siha cells. (G and H) Relative hsa_circ_0009035 expression by qRT-PCR in cells transduced with sh-NC, sh-hsa_circ_0009035, or sh-hsa_circ_0009035#2. *, *P < *0.05.

Using the online software CircView (http://gb.whu.edu.cn/CircView/), we found that hsa_circ_0009035, a 375-bp circRNA, was generated by the backsplicing of exons 15, 16, and 17 of RACGAP1 mRNA (Fig. 1A). The backsplicing junction was validated by Sanger sequencing (Fig. 1A). To investigate the involvement of hsa_circ_0009035 in CC development, we used quantitative real-time PCR (qRT-PCR) (primer details shown in Fig. 1A) to evaluate its expression level in CC tissues and adjacent normal tissues. In contrast, hsa_circ_0009035 expression was remarkably upregulated in CC tissues (Fig. 1B). Moreover, the level of hsa_circ_0009035 in the radiation-resistant group was higher than that of the sensitive group (Fig. 1C). In agreement with the CC tissues, hsa_circ_0009035 was significantly upregulated in CC cell lines compared with normal cervical Ect1/E6E7 cells (Fig. 1D). To elucidate the stability of hsa_circ_0009035, we performed RNase R and actinomycin D assays. These data showed that hsa_circ_0009035 was resistant to RNase R, and RNase R treatment led to a striking reduction in the level of corresponding linear mRNA (Fig. 2A and B). Furthermore, the incubation of cells with actinomycin D caused a significant downregulation of RACGAP1 linear mRNA, and hsa_circ_0009035 level did not decrease in the assayed time frame (Fig. 2C and D). Additionally, subcellular localization analysis showed that hsa_circ_0009035 was present mainly in the cytoplasm of HeLa and Siha cells (Fig. 2E and F).

### Silencing of hsa_circ_0009035 weakened cell proliferation, migration, and invasion and enhanced apoptosis and radiosensitivity *in vitro*.

**FIG 3:**
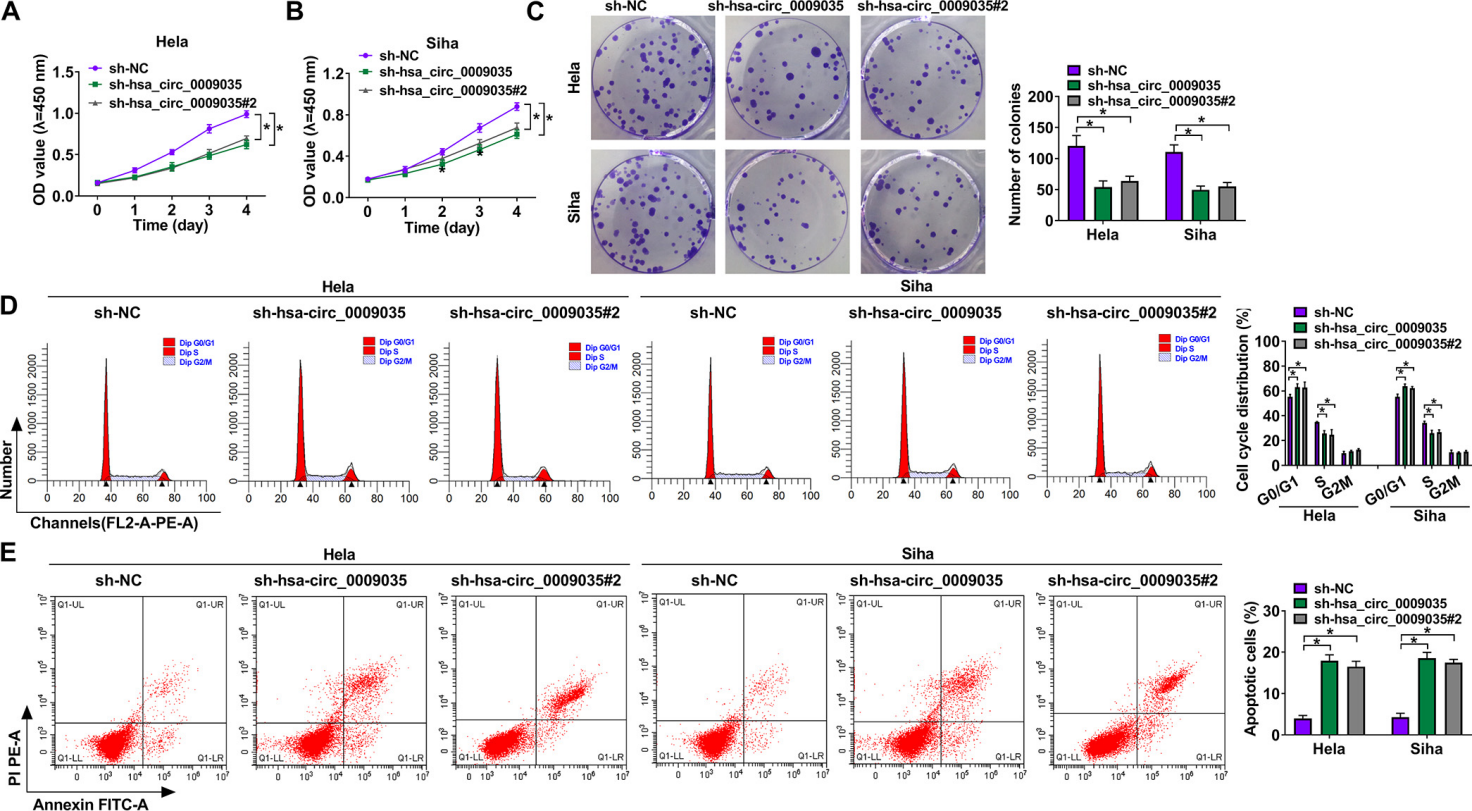
hsa_circ_0009035 silencing regulated CC cell proliferation and apoptosis *in vitro*. HeLa and Siha cells were stably transduced with sh-NC, sh-hsa_circ_0009035, or sh-hsa_circ_0009035#2. (A and B) Cell proliferation by CCK-8 assay. (C) Cell colony formation by colony formation assay. (D and E) Cell cycle progression and apoptosis by flow cytometry. *, *P < *0.05.

**FIG 4:**
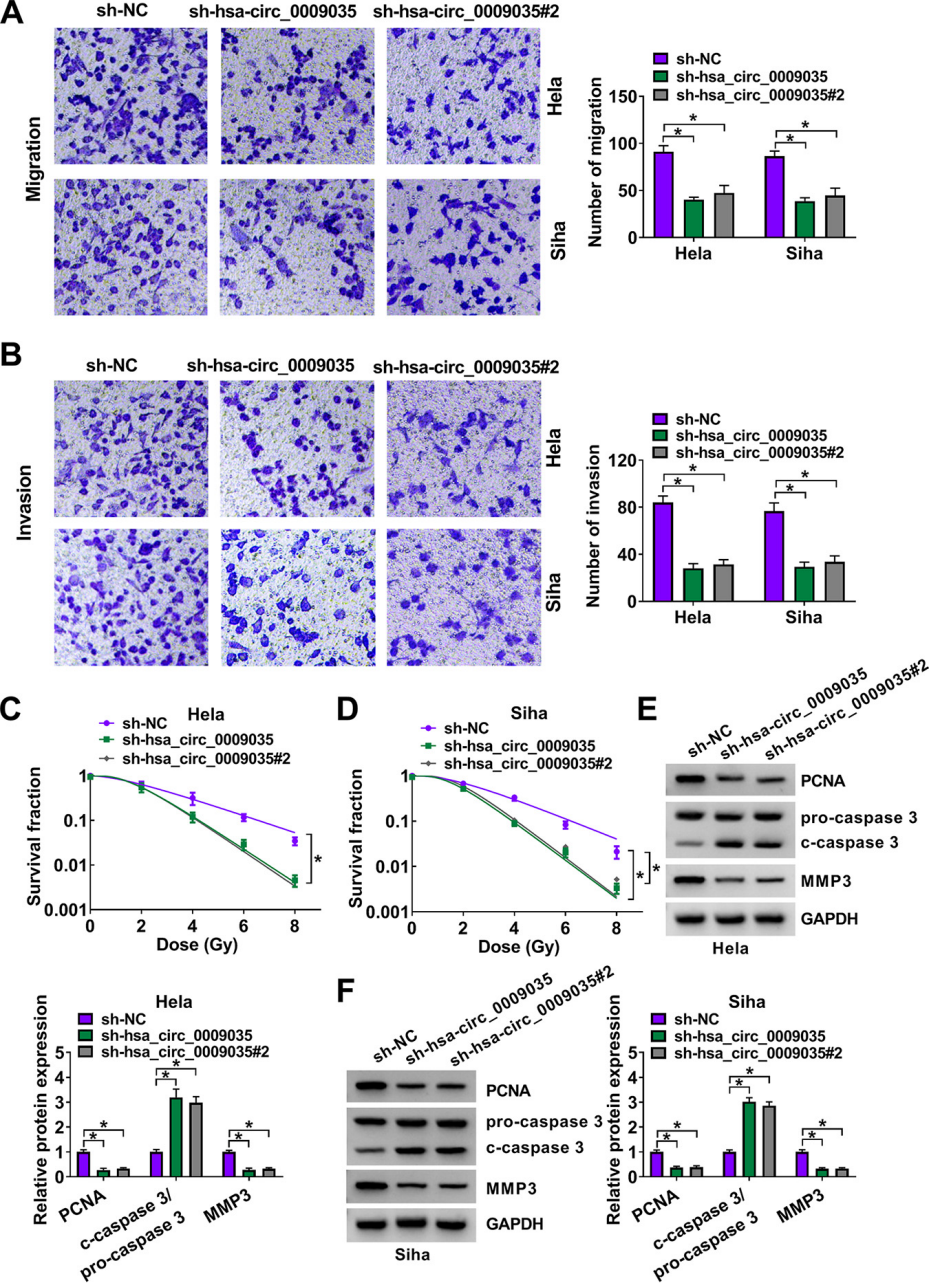
hsa_circ_0009035 silencing regulated CC cell migration, invasion, and radiosensitivity *in vitro*. HeLa and Siha cells were stably transduced with sh-NC, sh-hsa_circ_0009035, or sh-hsa_circ_0009035#2. (A and B) Cell migration and invasion by transwell assay. (C and D) Survival analysis by colony formation assay in transduced cells upon radiation (0, 2, 4, 6, and 8 Gy) exposure. (E and F) Protein levels of PCNA, c-caspase 3, procaspase 3, and MMP3 determined by Western blotting in transduced cells. *, *P < *0.05.

**FIG 5:**
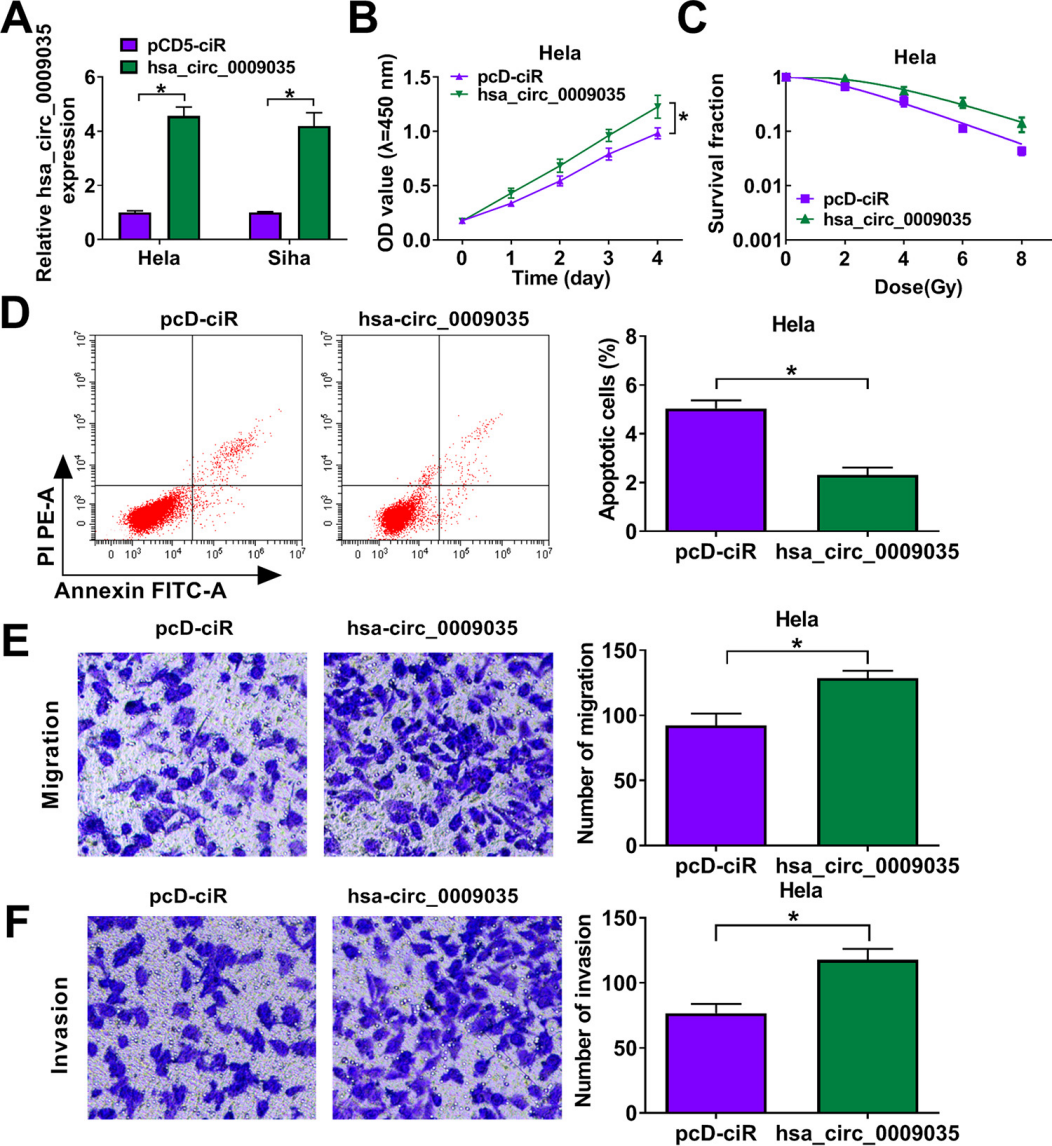
hsa_circ_0009035 overexpression regulated CC cell proliferation, apoptosis, migration, and invasion *in vitro*. HeLa and Siha cells were transfected with negative-control plasmid pcD-ciR or an hsa_circ_0009035 overexpression plasmid. (A) Relative hsa_circ_0009035 expression by qRT-PCR in transfected cells. (B and C) Cell proliferation by CCK-8 assay. (D) Cell apoptosis by flow cytometry. (E and F) Cell migration and invasion by transwell assay. *, *P < *0.05.

To directly test the functional role of hsa_circ_0009035 in CC progression, we carried out loss-of-function analyses *in vitro* with hsa_circ_0009035-shRNA lentiviruses (sh-hsa_circ_0009035 and sh-hsa_circ_0009035#2; short hairpin RNA [shRNA] details are shown in Fig. 1A). The introduction of sh-hsa_circ_0009035 or sh-hsa_circ_0009035#2 significantly reduced the expression of hsa_circ_0009035 but did not affect RACGAP1 mRNA level (Fig. 2G and H). Remarkably, the knockdown of hsa_circ_0009035 suppressed cell proliferation (Fig. 3A and B), colony formation (Fig. 3C), and cell cycle progression (Fig. 3D). Conversely, the silencing of hsa_circ_0009035 dramatically promoted cell apoptosis (Fig. 3E). Moreover, hsa_circ_0009035 silencing strongly repressed cell migration (Fig. 4A) and invasion (Fig. 4B). We also determined whether hsa_circ_0009035 could influence the radiosensitivity of CC cells *in vitro*. As expected, when cells were transduced with sh-hsa_circ_0009035 or sh-hsa_circ_0009035#2, the cell survival fraction was significantly reduced upon radiation exposure (Fig. 4C and D), demonstrating that hsa_circ_0009035 silencing enhanced cell radiosensitivity. Additionally, hsa_circ_0009035 knockdown led to a remarkable increase in the level of apoptosis-related protein cleaved caspase 3 (c-caspase 3) and a striking decrease in the expression of the proliferating marker PCNA and the invasion-related protein MMP3 in the two CC cell lines (Fig. 4E and F). Additionally, we overexpressed hsa_circ_0009035 in HeLa cells with an overexpression plasmid (Fig. 5A), and we found that the forced expression of hsa_circ_0009035 enhanced cell proliferation, migration, and invasion and suppressed apoptosis and radiosensitivity (Fig. 5B to F).

### hsa_circ_0009035 directly targeted miR-889-3p.

**FIG 6:**
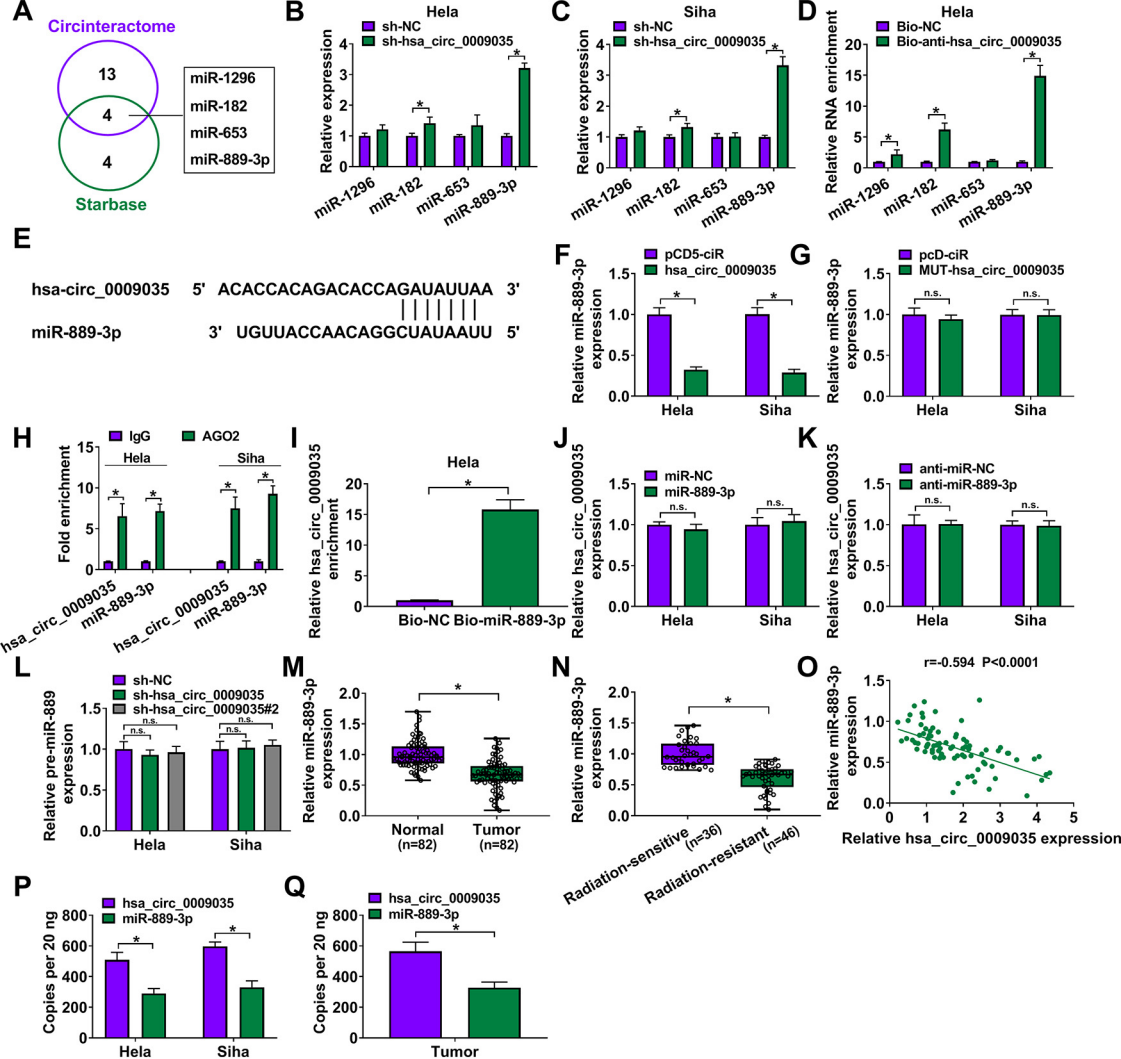
hsa_circ_0009035 in CC cells directly targeted miR-889-3p by binding to miR-889-3p. (A) Venn diagram of the putative miRNAs of hsa_circ_0009035 predicted by Circinteractome and Starbase softwares. (B and C) The expression levels of miR-1296, miR-182, miR-653, and miR-889-3p by qRT-PCR in sh-NC-infected or sh-hsa_circ_0009035-transduced HeLa and Siha cells. (D) RNA pulldown assays in HeLa cells using Bio-anti-hsa_circ_0009035 or Bio-NC. (E) Sequence of miR-889-3p and the putative miR-889-3p-binding sequence within hsa_circ_0009035. (F and G) Relative miR-889-3p expression determined by qRT-PCR in HeLa and Siha cells transfected with negative-control plasmid pCD5-ciR, an hsa_circ_0009035-overexpressing plasmid (hsa_circ_0009035), or a mutant hsa_circ_0009035-overexpressing plasmid (MUT-hsa_circ_0009035). (H) RIP assay in HeLa and Siha cells using IgG or AGO2 antibody. (I) RNA pulldown assays in HeLa cells using Bio-NC or Bio-miR-889-3p. (J and K) qRT-PCR analysis of hsa_circ_0009035 expression in cells transfected with miR-NC mimic, miR-889-3p mimic, anti-miR-889-3p, or anti-miR-NC. (L) The level of pre-miR-899 by qRT-PCR in cells transduced with sh-NC, sh-hsa_circ_0009035, or sh-hsa_circ_0009035#2. Relative miR-889-3p expression by qRT-PCR in 82 pairs of CC tissues and adjacent normal tissues (M) and CC tissues from 36 primary patients (defined as radiation-sensitive CC) and 46 recurrent patients after radiation treatment (defined as radiation-resistant CC) (N). (O) Correlation between miR-889-3p and hsa_circ_0009035 expression levels in CC tissues using the Spearman test. (P and Q) Copy numbers of hsa_circ_0009035 and miR-889-3p in HeLa and Siha cells and 5 case CC tissues. *, *P < *0.05.

To elucidate the mechanism by which hsa_circ_0009035 modulated CC progression and radiosensitivity, we used computer algorithms to help identify its targeted miRNAs. Interestingly, the prediction programs Circinteractome and Starbase showed that hsa_circ_0009035 harbored putative regions that matched the seed sequences of miR-1296, miR-182, miR-653, and miR-889-3p (Fig. 6A). In contrast, miR-889-3p was the most significantly upregulated miRNA in the sh-hsa_circ_0009035-transduced CC cells (Fig. 6B and C), and a biotinylated antisense oligomer (Bio-anti-hsa_circ_0009035) spanning the junction of hsa_circ_0009035 led to a remarkable increase in the enrichment level of miR-889-3p in HeLa cells (Fig. 6D). Thus, we selected miR-889-3p for further analysis and found a binding sequence for miR-889-3p within hsa_circ_0009035 (Fig. 6E).

We then determined whether hsa_circ_0009035 could influence miR-889-3p expression. As expected, the forced expression of hsa_circ_0009035 caused a significant downregulation of miR-889-3p level in both cell lines (Fig. 6F). However, the overexpression of mutant hsa_circ_0009035 lacking the miR-889-3p binding sites did not reduce the level of miR-889-3p (Fig. 6G), reinforcing that hsa_circ_0009035 regulated miR-889-3p via the binding site. RNA immunoprecipitation (RIP) assays revealed that the enrichment levels of hsa_circ_0009035 and miR-889-3p were synchronously increased in the presence of AGO2 antibody (Fig. 6H). RNA pulldown assays showed that the hsa_circ_0009035 enrichment level was markedly augmented by a biotin-labeled miR-889-3p mimic (Bio-miR-889-3p) (Fig. 6I), whereas the endogenous expression of hsa_circ_0009035 was not affected by an miR-889-3p mimic or anti-miR-889-3p transfection (Fig. 6J and K). Our data also showed that the silencing of hsa_circ_0009035 did not affect the level of pre-miR-889 in HeLa and Siha cells (Fig. 6L). In addition, miR-889-3p level was significantly downregulated in CC tissues compared with matched normal tissues, and miR-889-3p expression was lower in radiation-resistant tissues than that in sensitive tissues (Fig. 6M and N). More intriguingly, a strong inverse correlation between miR-889-3p and hsa_circ_0009035 expression levels was found in CC tissues (Fig. 6O). Analysis of the absolute expression of hsa_circ_0009035 and miR-889-3p in CC cells and tissues showed that the copy number of hsa_circ_0009035 is higher than that of miR-889-3p in CC tissues and HeLa and Siha cells (Fig. 6P and Q), suggesting that hsa_circ_0009035 was likely to bind to miR-889-3p, thereby liberating target mRNAs.

### HOXB7 in CC cells was directly targeted and inhibited by miR-889-3p.

**FIG 7:**
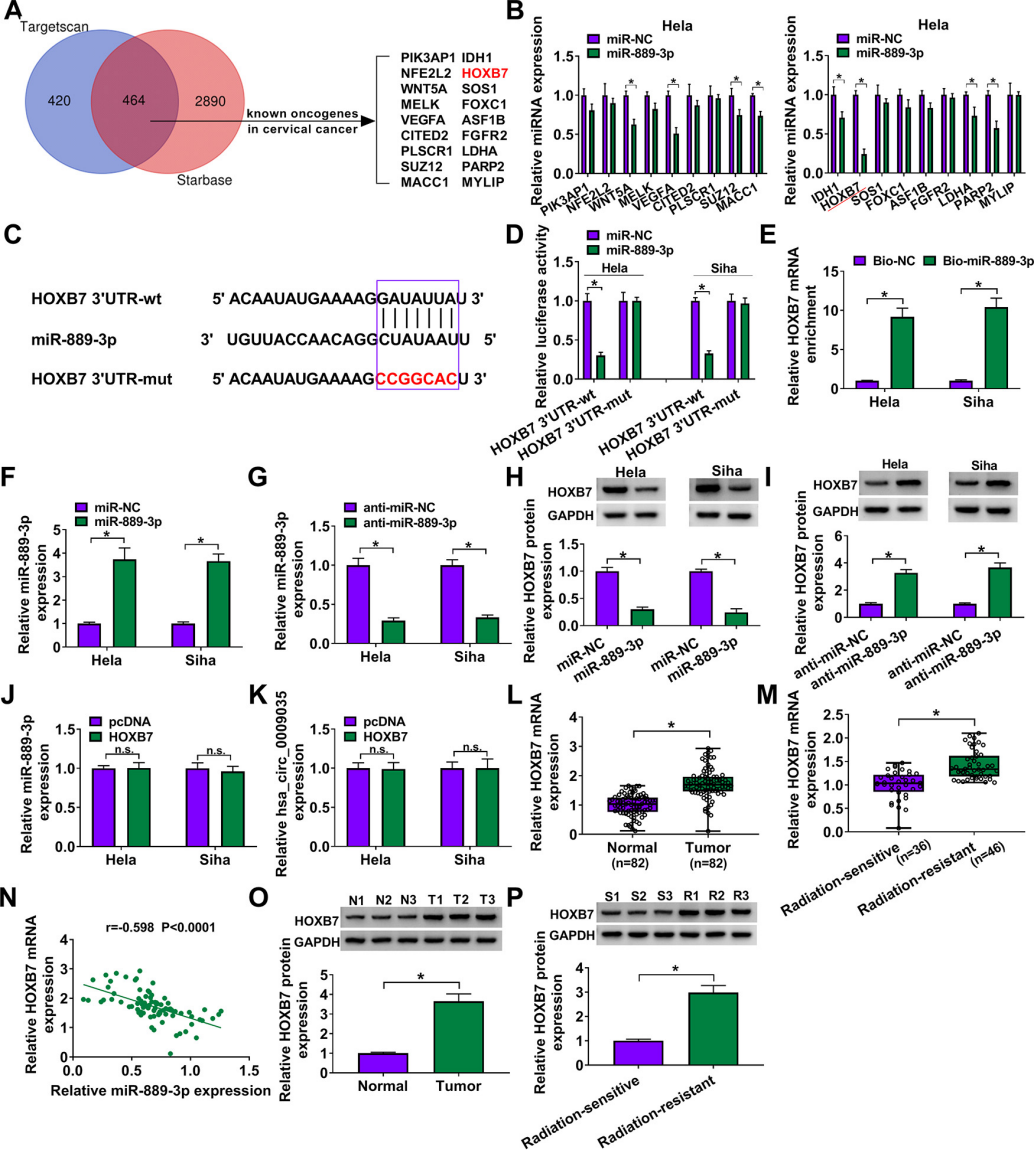
HOXB7 was a direct target of miR-889-3p in CC cells. (A) Venn diagram showing the putative targets of miR-889-3p predicted by Targetscan and Starbase softwares. (B) qRT-PCR analysis of genes in HeLa cells transfected with the miR-NC mimic or miR-889-3p mimic. (C) Schematic of the target sequence for miR-889-3p identified by Starbase software and the mutant of the seed region. (D) Dual-luciferase reporter assays in both HeLa and Siha cells. (E) RNA pulldown assays in HeLa and Siha cells using Bio-NC or Bio-miR-889-3p. Relative miR-889-3p expression by qRT-PCR in cells transfected with an miR-NC mimic, an miR-889-3p mimic (F), anti-miR-NC, or anti-miR-889-3p (G). HOXB7 protein level determined by Western blotting in cells transfected with an miR-NC mimic, an miR-889-3p mimic (H), anti-miR-NC, or anti-miR-889-3p (I). (J and K) qRT-PCR analysis of miR-889-3p and hsa_circ_0009035 expression levels in cells transfected with a negative-control plasmid (pcDNA) or a HOXB7-overexpressing plasmid (HOXB7). Relative HOXB7 expression determined by qRT-PCR in 82 pairs of CC tissues and adjacent normal tissues (L), CC tissues from 36 primary patients (defined as radiation-sensitive CC) and 46 recurrent patients after radiation treatment (defined as radiation-resistant CC) (M). (N) Correlation between HOXB7 mRNA and miR-889-3p expression levels in CC tissues using the Spearman test. HOXB7 protein expression in 3 pairs of CC tissues and adjacent normal tissues (O), CC tissues from 3 primary patients (defined radiation-sensitive CC) and 3 recurrent patients after radiation treatment (defined radiation-resistant CC) (P). *, *P < *0.05.

To further understand the role of miR-889-3p, we performed a detailed analysis for its molecular targets using Targetscan and Starbase online algorithms. Of the genes that overlapped between the two algorithms, we selected some that were identified as oncogenic drivers in CC (Fig. 7A). Because HOXB7 was the most significantly downregulated gene in miR-889-3p-overexpressing HeLa cells (Fig. 7B), we selected it for further analysis. The predicted data showed a putative complementary sequence for miR-889-3p within the HOXB7 3′ untranslated region (UTR) (Fig. 7C). To confirm this, we constructed HOXB7 3′ UTR wild-type (HOXB7 3′UTR-wt) and mutant (HOXB7 3′UTR-mut) luciferase reporters and analyzed them by luciferase activity. The transfection of the miR-889-3p mimic significantly reduced the luciferase activity of HOXB7 3′UTR-wt but barely affected the luciferase activity of HOXB7 3′UTR-mut (Fig. 7D). Moreover, RNA pulldown assays showed that the enrichment level of HOXB7 mRNA was dramatically augmented by a biotin-labeled miR-889-3p mimic (Bio-miR-889-3p) (Fig. 7E). We then elucidated whether miR-889-3p could modulate HOXB7 expression. The transfection efficiencies of the miR-889-3p mimic and anti-miR-889-3p were determined by qRT-PCR (Fig. 7F and G). Remarkably, HOXB7 protein level was reduced by miR-889-3p overexpression and increased as a result of miR-889-3p downregulation in both cell lines (Fig. 7H and I). Our data also showed that the overexpression of HOXB7 did not affect the levels of endogenous miR-889-3p and hsa_circ_0009035 in the two cell lines (Fig. 7J and K). Additionally, HOXB7 mRNA expression was significantly upregulated in CC tissues, and the radiation-resistant tissues had a higher HOXB7 level than the sensitive CC tissues (Fig. 7L and M). Importantly, a strong inverse correlation between HOXB7 mRNA and miR-889-3p expression levels in CC tissues was found (Fig. 7N). Furthermore, in line with mRNA expression, HOXB7 protein was strikingly overexpressed in CC tissues and radiation-resistant CC tissues compared to their counterparts (Fig. 7O and P).

### Forced expression of miR-889-3p regulated cell proliferation, migration, invasion, apoptosis, and radiosensitivity *in vitro* by downregulating HOXB7.

**FIG 8:**
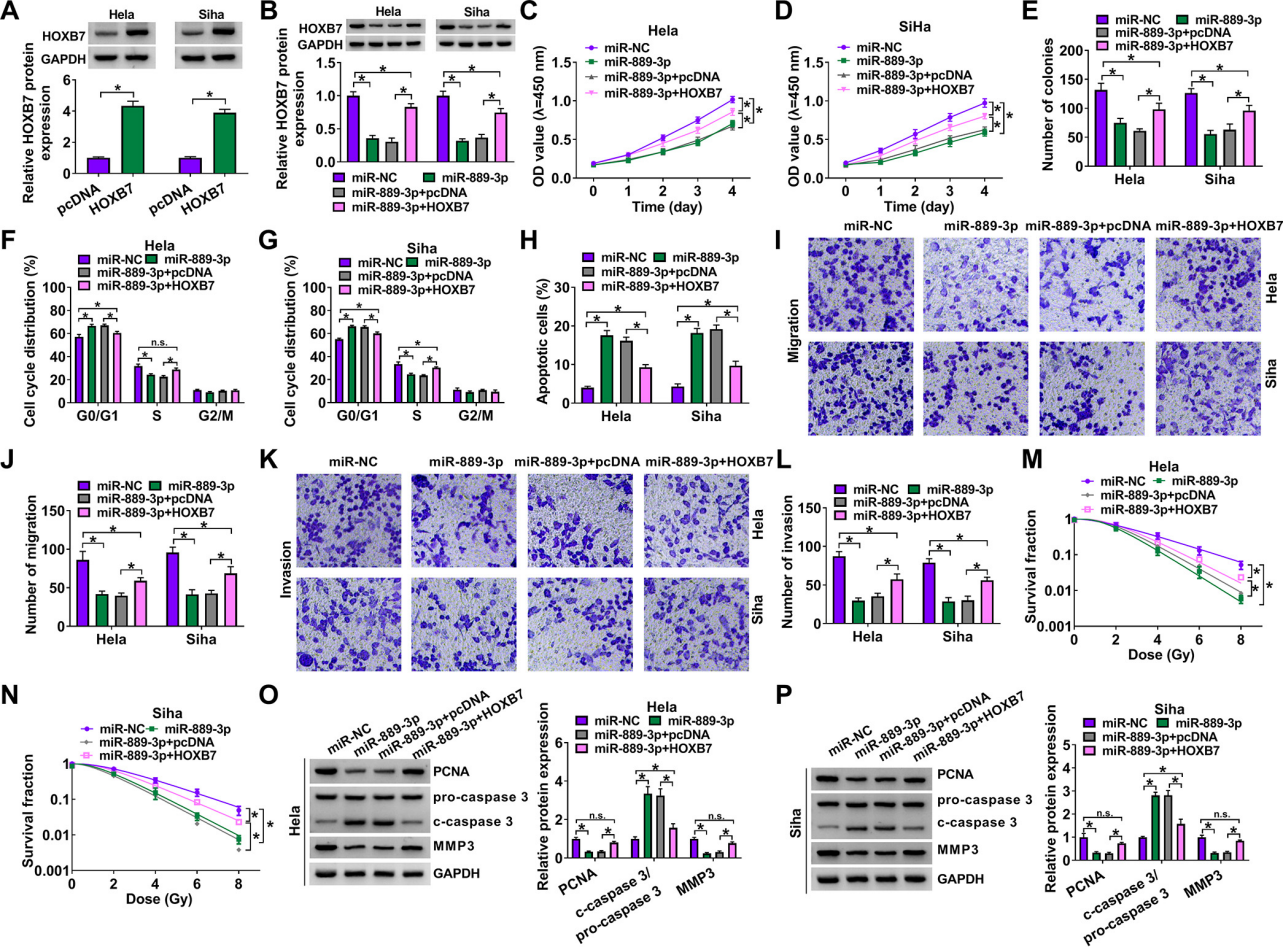
The effects of miR-889-3p overexpression on CC progression and radiosensitivity *in vitro* were mediated by HOXB7. (A) Relative HOXB7 protein level determined by Western blotting in cells transfected with a negative-control plasmid (pcDNA) or a HOXB7-overexpressing plasmid (HOXB7). HeLa and Siha cells were transfected with an miR-NC mimic, an miR-899-3p mimic, an miR-899-3p mimic plus pcDNA, or an miR-899-3p mimic plus HOXB7, followed by the determination of HOXB7 protein level by Western blotting (B), cell proliferation by CCK-8 assay (C and D), analysis of cell colony formation by colony formation assay (E), analysis of cell cycle progression and apoptosis by flow cytometry (F to H), analysis of cell migration and invasion by transwell assay (I to L), determination of cell survival fraction by colony formation upon radiation exposure (M and N), and measurement of the levels of PCNA, c-caspase 3, procaspase 3, and MMP3 by Western blotting (O and P). *, *P < *0.05.

To determine whether HOXB7 was a functional target of miR-889-3p in modulating CC progression and radiosensitivity, we upregulated the HOXB7 level in miR-889-3p-overexpressing CC cells. The transfection efficiency of a HOXB7-overexpressing plasmid was gauged by Western blotting (Fig. 8A). Remarkably, the transfection of the HOXB7-overexpressing plasmid abolished the reduction of miR-889-3p overexpression on HOXB7 protein level in both cell lines (Fig. 8B). In contrast, the forced level of miR-889-3p significantly weakened cell proliferation (Fig. 8C and D), colony formation (Fig. 8E), cell cycle progression (Fig. 8F and G), and enhanced cell apoptosis (Fig. 8H), as well as suppressing cell migration (Fig. 8I and J) and invasion (Fig. 8K and L). Furthermore, miR-889-3p overexpression dramatically reduced cell survival fraction upon radiation exposure (Fig. 8M and N). In addition, the forced miR-889-3p level resulted in increased c-caspase 3 expression and decreased levels of PCNA and MMP3 in the two CC cell lines (Fig. 8O and P). However, these functional regulatory effects of miR-889-3p overexpression were significantly abrogated by the restored expression of HOXB7 in both cell lines (Fig. 8C to P).

### hsa_circ_0009035 modulated HOXB7 expression and CC progression and radiosensitivity *in vitro* by targeting miR-889-3p.

**FIG 9:**
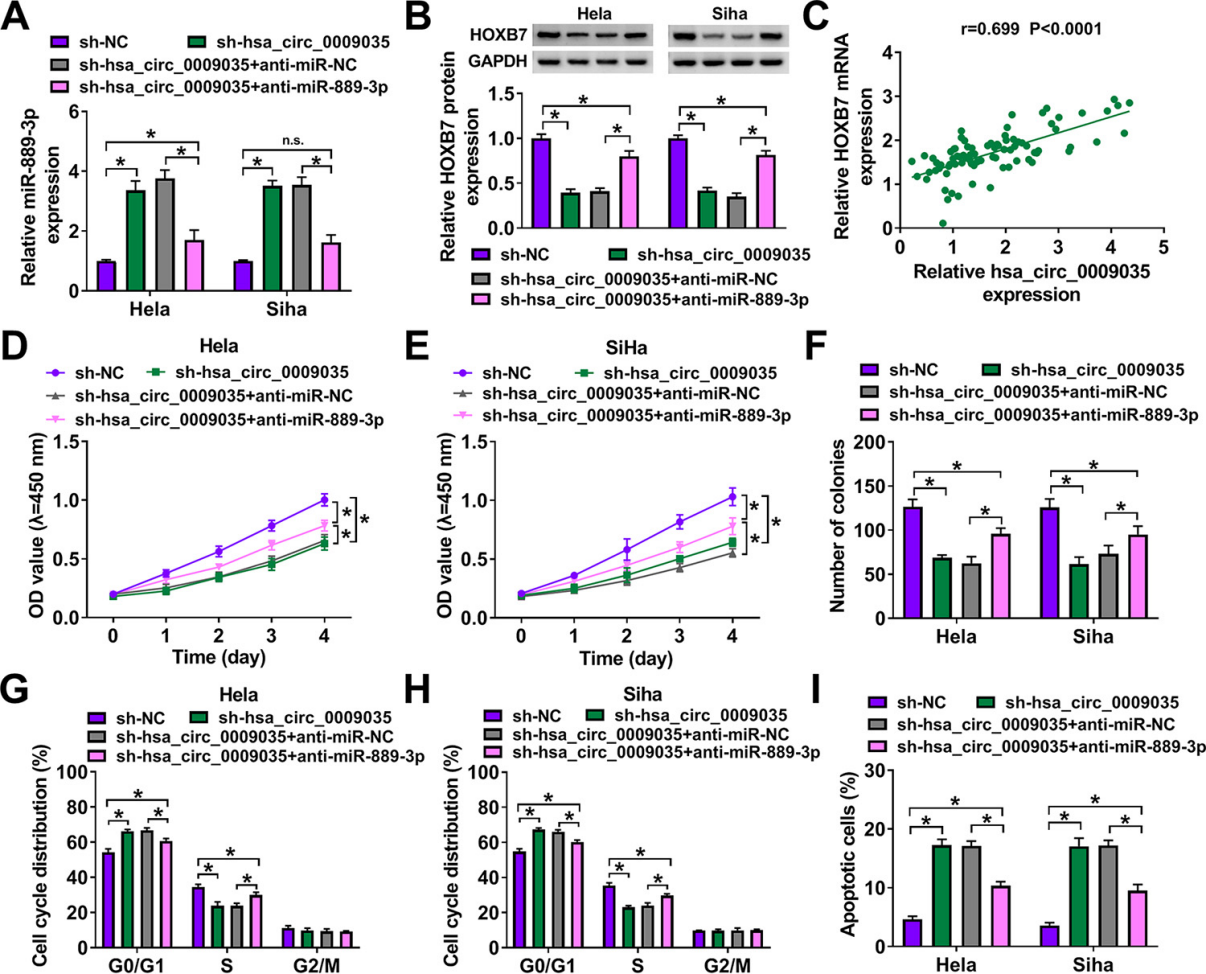
hsa_circ_0009035 modulated HOXB7 expression and CC progression *in vitro* by miR-889-3p. Expression levels of miR-889-3p (A) and HOXB7 protein (B) in sh-NC-infected or sh-hsa_circ_0009035-transduced cells transfected with or without anti-miR-NC or anti-miR-889-3p. (C) Correlation between HOXB7 mRNA and hsa_circ_0009035 expression levels in CC tissues using the Spearman test. sh-NC-infected or sh-hsa_circ_0009035-transduced HeLa and Siha cells were transfected with or without anti-miR-NC or anti-miR-889-3p, followed by the assessment of cell proliferation by CCK-8 assay (D and E), cell colony formation by colony formation assay (F), and cell cycle progression and apoptosis by flow cytometry (G to I). *, *P < *0.05.

We then determined whether hsa_circ_0009035 could modulate HOXB7 expression by miR-889-3p. In contrast, the transfection of anti-miR-889-3p prominently abrogated the increase of miR-899-3p expression of hsa_circ_0009035 silencing in both cell lines (Fig. 9A). As expected, the silencing of hsa_circ_0009035 led to a prominent reduction in the level of HOXB7 protein, and this effect was significantly abolished by miR-889-3p downregulation (Fig. 9B), suggesting that hsa_circ_0009035 modulated HOXB7 expression by regulating miR-889-3p. Interestingly, we found a strong positive correlation between HOXB7 mRNA and hsa_circ_0009035 levels in CC tissues (Fig. 9C).

**FIG 10:**
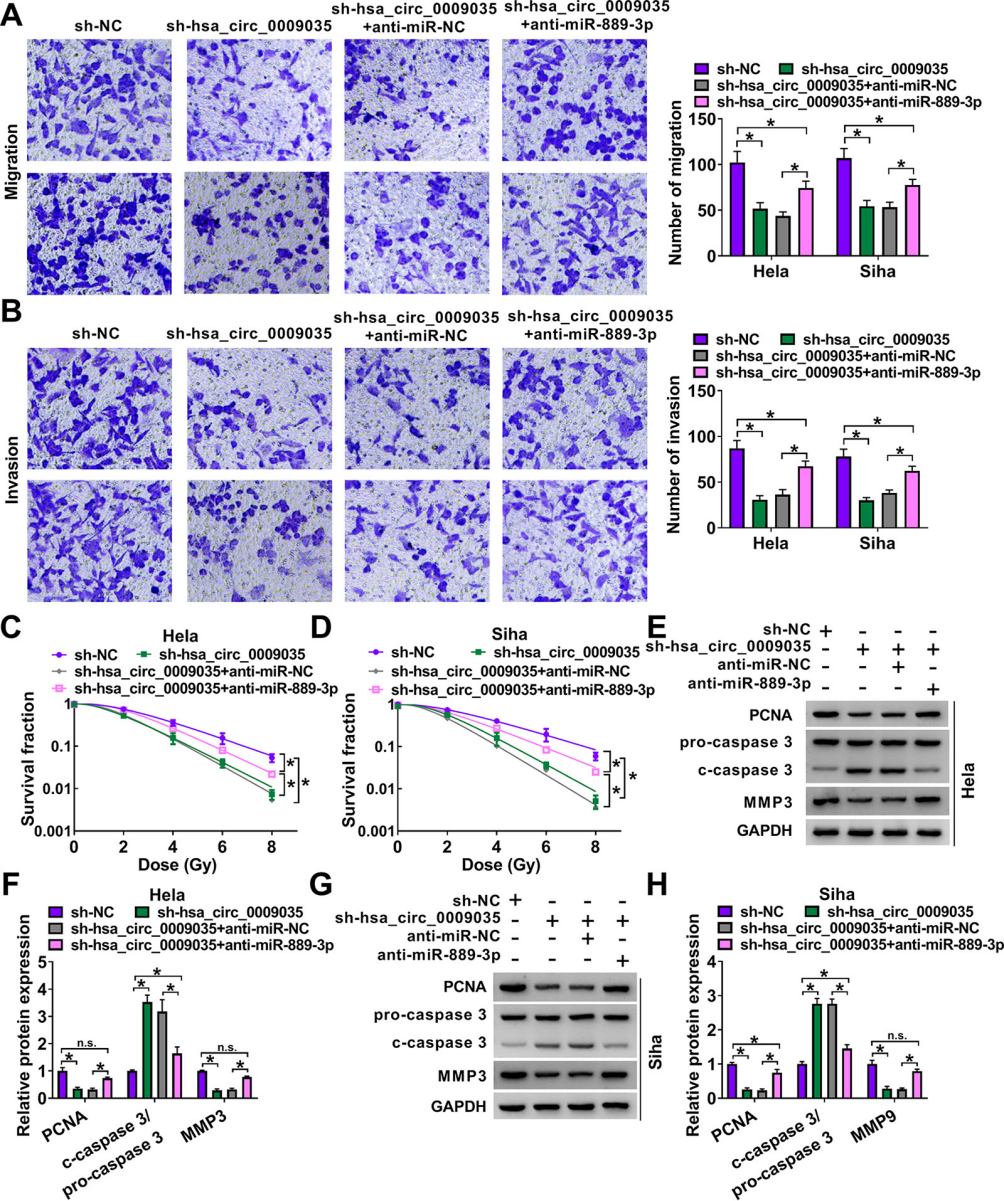
hsa_circ_0009035 modulated CC migration, invasion, and radiosensitivity *in vitro* by miR-889-3p. sh-NC-infected or sh-hsa_circ_0009035-transduced HeLa and Siha cells were transfected with or without anti-miR-NC or anti-miR-889-3p, followed by the assessment of cell migration and invasion by transwell assay (A and B), cell survival fraction by colony formation assay upon radiation exposure (C and D), and levels of PCNA, c-caspase 3, procaspase 3, and MMP3 by Western blotting (E to H). *, *P < *0.05.

A crucial question was whether miR-889-3p could work as a downstream effector of hsa_circ_0009035 in regulating CC progression and radiosensitivity. To address this, we carried out rescue experiments in the two CC cell lines. These results revealed that the reduced level of miR-889-3p remarkably abrogated sh-hsa_circ_0009035-mediated antiproliferation (Fig. 9D and E), anti-colony formation (Fig. 9F), cell cycle arrest (Fig. 9G and H), proapoptosis (Fig. 9I), antimigration (Fig. 10A), and anti-invasion (Fig. 10B). Moreover, the downregulation of miR-889-3p significantly reversed the reduction of survival fraction of hsa_circ_0009035 silencing upon radiation exposure (Fig. 10C and D). Additionally, the reduced level of miR-889-3p abolished the impact of hsa_circ_0009035 silencing on PCNA, c-caspase 3, and MMP3 levels in the two CC cell lines (Fig. 10E to H).

### hsa_circ_0009035 regulated tumor growth *in vivo*.

**FIG 11:**
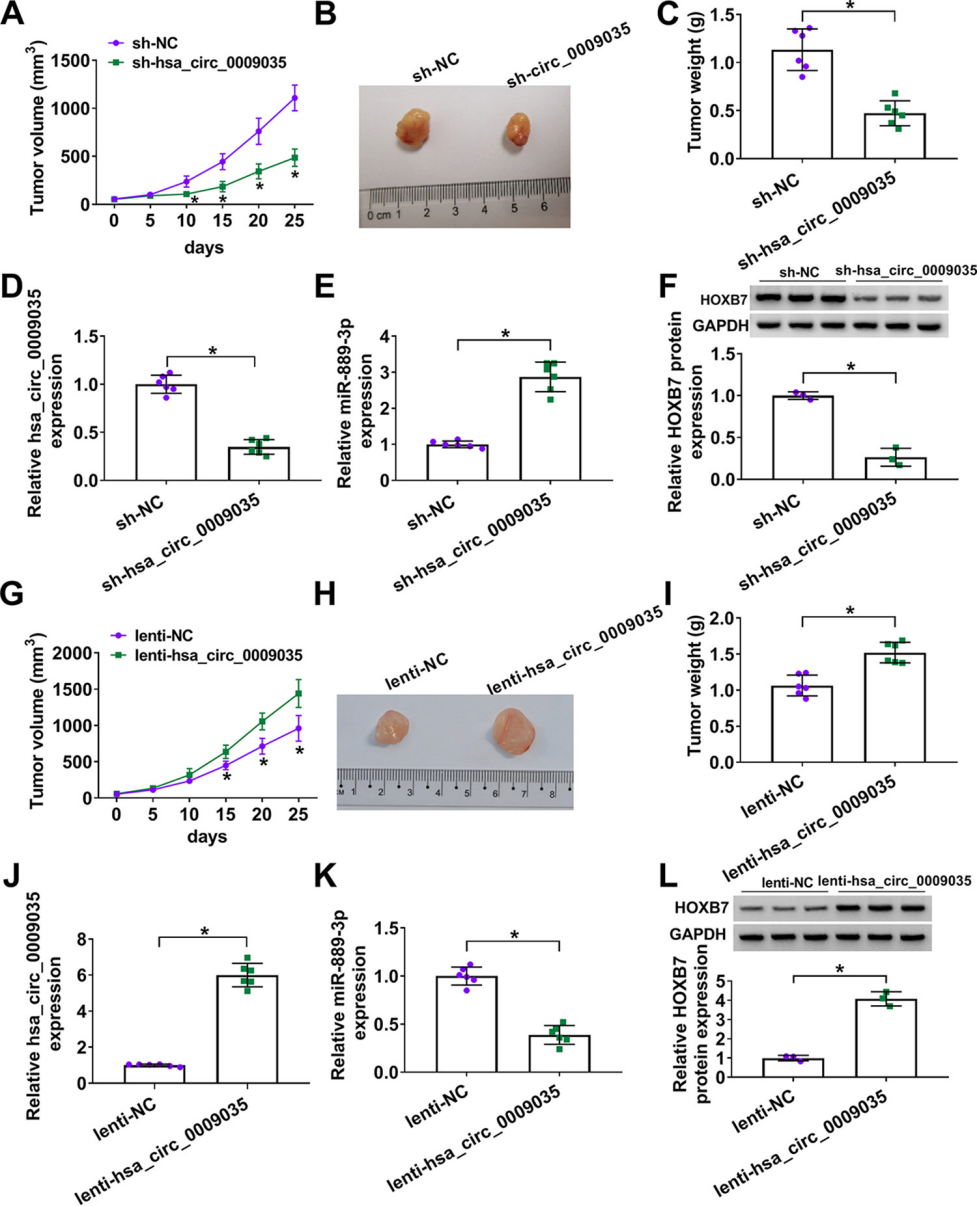
hsa_circ_0009035 regulated tumor growth *in vivo*. (A) Growth curves of the xenograft tumors formed by sh-NC-infected or sh-hsa_circ_0009035-transduced HeLa cells (*n* = 6 per group). Representative images (B), tumor average weight (C), hsa_circ_0009035 (D) and miR-889-3p (E) levels determined by qRT-PCR, and HOXB7 protein expression determined by Western blotting (F) of the xenograft tumors formed by HeLa cells infected with sh-NC or sh-hsa_circ_0009035, on day 25 after subcutaneous injection (*n* = 6 per group). (G) Growth curves of the xenograft tumors formed by lenti-NC-infected or lenti-hsa_circ_0009035-transduced HeLa cells (*n* = 6 per group). Representative images (H), tumor average weight (I), hsa_circ_0009035 (J), and miR-889-3p (K) levels determined by qRT-PCR, and HOXB7 protein expression determined by Western blotting (L) of the xenograft tumors formed by HeLa cells infected with lenti-NC or lenti-hsa_circ_0009035, on day 25 after subcutaneous injection (*n* = 6 per group). *, *P < *0.05.

We next asked whether hsa_circ_0009035 could modulate tumor growth *in vivo*. To address this, we established the xenograft mouse model using sh-hsa_circ_0009035-transduced or lenti-hsa_circ_0009035-infected HeLa cells by subcutaneous injection. In contrast, sh-hsa_circ_0009035 transduction significantly weakened tumor growth (Fig. 11A to C). qRT-PCR analysis showed that hsa_circ_0009035 was downregulated in sh-hsa_circ_0009035-transduced HeLa tumors (Fig. 11D). Moreover, the knockdown of hsa_circ_0009035 led to an increase in the expression of miR-889-3p and a decrease in the level of HOXB7 protein (Fig. 11E and F). Conversely, the transduction of lenti-hsa_circ_0009035 remarkably enhanced tumor growth (Fig. 11G to I). hsa_circ_0009035 was highly expressed in lenti-hsa_circ_0009035-transduced xenograft tumors (Fig. 11J). Furthermore, the overexpression of hsa_circ_0009035 led to a reduction in the expression of miR-889-3p and a clear increase in the level of HOXB7 protein (Fig. 11K and L).

## DISCUSSION

**FIG 12:**
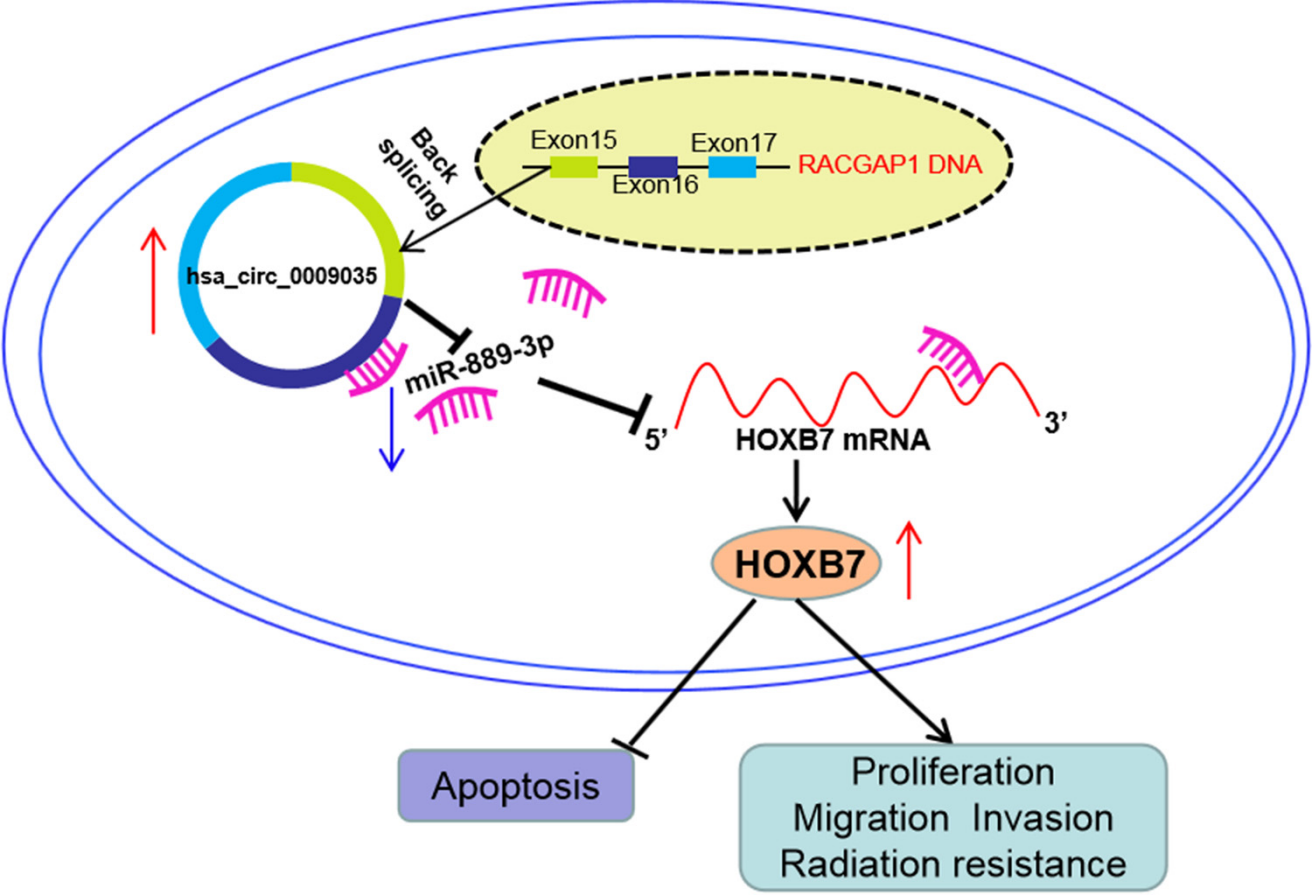
Schematic model of the hsa_circ_0009035/miR-889-3p/HOXB7 axis in CC progression and radiosensitivity. In CC, hsa_circ_0009035 was overexpressed and the increased level of hsa_circ_0009035 reduced the miR-889-3p level. Then, the downregulation of miR-889-3p increased HOXB7 expression. Finally, HOXB7 overexpression promoted cell proliferation, migration, invasion, and radiation resistance and suppressed cell apoptosis, thereby enhancing CC progression and radiation resistance.

Recently, the altered expression of circRNAs has been demonstrated to influence CC carcinogenesis and chemoresistance (6, 12, 17). Although a large number of circRNAs were discovered to be differentially expressed in radioresistant HeLa cells (13), the precise actions of these circRNAs in CC radioresistance are still elusive. Here, we report, for the first time, the functional actions and molecular determinant of hsa_circ_0009035 in CC progression and radioresistance (Fig. 12).

A recent study uncovered the promotional impact of circRACGAP1 on gastric cancer cell sensitivity to apatinib through the modulation of the miR-3657/ATG7 axis (18). As a member of circRACGAP1, hsa_circ_0009035, an upregulated circRNA in radioresistant HeLa cells, was illuminated the crucial involvement in CC radioresistance (13). Our results indicated that hsa_circ_0009035 was upregulated in CC and associated with the radioresistance of CC patients, implying its potential clinical significance as a diagnostic biomarker. MMP3, a key member of the MMP family, has been implicated in CC cell migration and invasion (19). Our results showed that hsa_circ_0009035 regulated CC progression and radiosensitivity *in vitro*, as well as modulating tumor growth *in vivo*. Additionally, as previously reported for other circRNAs (20, 21), hsa_circ_0009035 were unusually stable and resistant to RNase R, which was attributed to their covalently closed loop structures with neither 5′ caps nor 3′ polyadenylation tails (22).

Several previous reports had demonstrated the conflicting roles of miR-889-3p in human carcinogenesis (23–25). These contradictory conclusions might be partially due to the different tumor types in these reports, where miR-889-3p exhibited an antitumor property in non-small cell lung cancer (25) and functioned as a tumor promoter in esophageal squamous cell carcinoma (23) and osteosarcoma (24). Interestingly, miR-889-3p was identified as a strong tumor suppressor in CC (15, 26). Our results extended this by determining the regulation of miR-889-3p on CC radiosensitivity. Moreover, we first discovered that hsa_circ_0009035 directly bound to miR-889-3p, and miR-889-3p was a functionally important mediator of hsa_circ_0009035 in modulating CC progression and radioresistance *in vitro*. Previous work also discovered the potential involvement of miR-889-3p in cell survival of radiation in nervous system cancer (27).

HOXB7, a member of the HOX gene family, has been shown as a strong oncogene in various human cancers, such as hepatocellular carcinoma, breast cancer, and osteosarcoma (28–30). Here, we first identified the role of hsa_circ_0009035 as a posttranscriptional regulator of HOXB7 expression by targeting miR-889-3p. Furthermore, for the first time, we showed that HOXB7 was a functional target of miR-889-3p in modulating CC progression and radioresistance *in vitro*. Similarly, How et al. reported that miR-196b hindered CC development by targeting HOXB7 (16). Previous studies also reported that several other miRNAs, such as miR-377 and miR-384, impacted human carcinogenesis by directly targeting HOXB7 (31, 32). The circRNA/miRNA/mRNA regulatory networks are intricate, and a circRNA can act as an inhibitor of many miRNAs (33). There may be alternative miRNA/mRNA axes that remain to be uncovered in the modulation of hsa_circ_0009035 in CC. Future studies will build on the findings by identifying precisely how the novel network modulates CC progression and radioresistance *in vitro* and *in vivo*.

In summary, our findings identify hsa_circ_0009035 as an important modulator in CC progression and radioresistance at least in part by targeting the miR-889-3p/HOXB7 axis. To our knowledge, this is the first report of hsa_circ_0009035 in human carcinogenesis, pointing to its significance as a potential therapeutic target for CC treatment.

## MATERIALS AND METHODS

### Human specimens and cells.

Specimen tissues, including cancer tissues and adjacent healthy cervical tissues, were obtained from 82 consecutive patients with CC (average age, 46.2 ± 8.1; 36 patients with primary CC and 46 with recurrent CC after radiation treatment) who underwent surgical resection at The First Affiliated Hospital of University of South China. All specimens were stored at −80°C until quantitative real-time PCR (qRT-PCR) analysis for the expression levels of hsa_circ_0009035, miR-889-3p, and HOXB7. The human study project was approved by the Ethics Committee of The First Affiliated Hospital of University of South China, and all patients gave written informed consent.

Human ectocervical Ect1/E6E7 cells and two CC cells (HeLa and Siha) were obtained from the American Type Culture Collection (ATCC, Rockville, MD, USA). HeLa and Siha cells were cultivated in accordance with the standard protocols provided by ATCC. Ect1/E6E7 cells were propagated in keratinocyte serum-free medium (Gibco, Lucerne, Switzerland) as reported elsewhere (34).

### Generation of stable cell lines.

Lentiviruses encoding hsa_circ_0009035-shRNAs (sh-hsa_circ_0009035, 5′-CTATAATTTCTTTAGTGTCTA-3′, and sh-hsa_circ_0009035#2, 5′-ATTTCTTTAGTGTCTACTTCT-3′), the nontarget shRNA (sh-NC, 5′-TTAGTGTGATGAAGACAAC-3′), hsa_circ_0009035 (lenti-hsa_circ_0009035), and a scrambled control sequence (lenti-NC) were purchased from HanBio (Shanghai, China). HeLa and Siha cells at ∼60% confluence were infected by the lentiviruses in medium containing 6 μg/ml of Polybrene (Yesen, Shanghai, China). One day after transduction, the cells were seeded into 24-well plates in the presence of puromycin (2 μg/ml; Yesen) for selection for at least 96 h.

### Cell transfection.

The human hsa_circ_0009035 sequence, the hsa_circ_0009035 sequence lacking the mniR-889-3p binding sites, and a nontarget control sequence were synthesized by BGI (Shenzhen, China) and individually inserted into a pCD5-ciR vector (Geneseed, Guangzhou, China) opened with EcoRI and BamHI sites to produce an hsa_circ_0009035 overexpression plasmid, a mutant hsa_circ_0009035 overexpression plasmid (MUT-hsa_circ_0009035), and a negative-control plasmid (pCD5-ciR). Human HOXB7 (accession no. NM_004502.4; synthesized by BGI) was cloned into a pcDNA3.1 vector (Invitrogen, Saint-Aubin, France) with BamHI and XhoI restriction sites to construct HOXB7 overexpressing plasmid, and a nontarget pcDNA3.1 plasmid (pcDNA) was used as the negative-control plasmid. A mature miR-889-3p mimic (5′-UUAAUAUCGGACAACCAUUGU-3′), a mimic negative control (miR-NC mimic; 5′-ACGUGACACGUUCGGAGAATT-3′), an miR-889-3p inhibitor designed for miR-889-3p silencing (anti-miR-889-3p; 5′-ACAAUGGUUGUCCGAUAUUAA-3′), and the scrambled oligonucleotide control (anti-miR-NC; 5′-CAGUACUUUUGUGUAGUACAA-3′) were obtained from Ribobio (Guangzhou, China). HeLa and Siha cells at ∼60% confluence in 24-well plates were transiently transfected with 200 ng of plasmids and 50 nM oligonucleotides using Lipofectamine 3000 as recommended by the manufacturer (Invitrogen). Cells were harvested for further experiments after 48 h.

### RNA extraction and qRT-PCR.

Total RNA containing small RNA was prepared from cells and tissues with the RNAiso Plus (TaKaRa, Beijing, China) as per the accompanying instructions. For hsa_circ_0009035 and mRNAs qRT-PCR, reverse transcription (RT) was done using the PrimeScript RT kit (TaKaRa). For miRNAs qRT-PCR, cDNA was generated with a TaqMan microRNA RT kit (Applied Biosystems, Courtaboeuf, France) based on the protocols of the manufacturers. qRT-PCR analysis with designed primers (Table 1) was carried out in triplicate using a SYBR green kit (TaKaRa) on an Agilent 2100 Bioanalyzer (Agilent Technologies, Santa Clara, CA, USA). Human β-actin or U6 was used for normalization. Fold changes in gene expression levels were calculated by the 2^−ΔΔ^*^CT^* method (35).

**TABLE 1:** Sequences of qRT-PCR primers

Primer	Direction	Sequence (5′–3′)
hsa_circ_0009035	Forward	CATGATGGTGGAGCAAGAGA
Reverse	CATGGCAGCTATGCTGTTGT
RACGAP1 linear mRNA	Forward	CACTTGCAGAGAGTGGCTCA
Reverse	TCCAGAGGCAAGGAAAGCAG
HOXB7	Forward	CTCTGCCTCACGGAAAGACA
Reverse	AGTTTCCTGATTCAGTTCCCAGA
miR-889-3p	Forward	GCCGAGTTAATATCGGACAA
Reverse	CAGTGCGTGTCGTGGAGT
miR-182	Forward	GCCGAGTTTGGCAATGGTAG
Reverse	CAGTGCGTGTCGTGGAGT
miR-653	Forward	GCCGAGTTGAAACAATCTCT
Reverse	CTCAACTGGTGTCGTGGA
miR-1296	Forward	CGAGTTAGGGCCCTGGCT
Reverse	CTCAACTGGTGTCGTGGA
U6	Forward	CTCGCTTCGGCAGCACA
Reverse	AACGCTTCACGAATTTGCGT
β-actin	Forward	CTCGCCTTTGCCGATCC
Reverse	GGGGTACTTCAGGGTGAGGA

### RNase R assay.

RNase R treatment was carried out by adding 3 U of RNase R (Geneseed) to 1 μg of total RNA and incubating for 20 min at 37°C.

### Actinomycin D assay.

The actinomycin D test was implemented by adding actinomycin D (2 mg/ml; Sigma-Aldrich, Tokyo, Japan) to HeLa and Siha cells and incubating for 4, 8, 12, and 24 h at 37°C.

### Subcellular localization assay.

RNA extraction of cytoplasmic and nuclear fractions of HeLa and Siha cells was conducted using the cytoplasmic and nuclear RNA purification kit (Norgen Biotek, Thorold, ON, Canada) as per the accompanying protocols. Glyceraldehyde-3-phosphate dehydrogenase (GAPDH) and U6 served as cytoplasm and nucleus controls, respectively.

### Cell proliferation assay.

Transfected cells (1 × 10^3^ cells/well) were seeded in 100 μl of culture medium in 96-well plates and maintained at 37°C. After 1, 2, 3, and 4 days, the Cell Counting Kit-8 (CCK-8; Biotech, Nanjing, China) solution was added into per well at a dose of 10 μl. Following 3 h of incubation, the absorbance in each well was read at 450 nm using a microplate reader (Thermo Fisher Scientific, Paisley, UK).

### Colony formation and survival assays.

About 140 transfected cells were plated into each well of a 6-well plate and incubated at 37°C for 14 to 21 days. The plates were then stained with 0.5% crystal violet (Yesen), and the number of colonies containing 50 cells or more was evaluated under an inverted microscope (40× magnification; Leica, Wetzlar, Germany).

For survival analysis, 200 cells cultured on a 6-well plate were exposed to X-ray radiation for 8 h with cumulative dosage as indicated in the figures. Subsequently, colony formation assay was carried out as described above. Survival fraction in each experimental group was determined based on that of an untreated counterpart.

### Flow cytometry for cell cycle and apoptosis.

For cell cycle analysis, transfected cells (1 × 10^6^) were stained with propidium iodide (PI; Sigma-Aldrich) as per the accompanying guidance. For apoptosis analysis, transfected cells were incubated with annexin V-fluorescein isothiocyanate (FITC) (Invitrogen) and PI for 15 min in the dark. In both assays, 10,000 events in each sample were analyzed within 1 h. The apoptotic cells were calculated by summing the early (annexin V^+^ PI^+^) and late (annexin V^+^ PI^−^) apoptotic cells.

### Transwell migration and invasion assays.

Twenty-four-well transwell inserts (8-μm pores; Corning, Chengdu, China) were used for migration assays. Inserts precoated with Matrigel (Corning) were used for invasion assays. Transfected cells in serum-free medium were plated on the insert membranes at 2 × 10^4^ cells per well for migration assays and 1 × 10^5^ cells per well for invasion assays. The lower chamber was filled with the culture medium containing 10% serum. The cells that traversed the inserts were scored with a 100× magnification microscope after being stained with 0.5% crystal violet. Five random fields in each example were selected to get an average number of migrating or invading cells.

### Western blotting.

Total protein was prepared from cells and tissues with the xTractor buffer (TaKaRa) and then quantified by the Dc-assay kit (Bio-Rad, Munich, Germany). Samples (30 μg) were analyzed by electrophoresis with Mini-Protean TGX gels (Bio-Rad), and the resulting gels were electroblotted to nitrocellulose membranes (Schleicher & Schuell, Dassel, Germany). Proliferating cell nuclear antigen (PCNA; 13-3900; Invitrogen), pro-inactive caspase 3 (procaspase 3; ab32150; Abcam, Cambridge, UK), cleaved caspase 3 (c-caspase 3; ab49822; Abcam), matrix metalloproteinase 9 (MMP9; MA5-15886; Invitrogen), HOXB7 (40-2000; Invitrogen), and GAPDH (39-8600; Invitrogen) antibodies were selected as primary antibodies, and anti-mouse or anti-rabbit IgG conjugated with horseradish peroxidase (ab6789 or ab97051; Abcam) was used as the secondary antibody. Blots were detected with the enhanced chemiluminescence (Bio-Rad), and the intensities of the bands were analyzed with ImageJ software (National Institutes of Health, Bethesda, MD, USA).

### Bioinformatics.

The directly interacting miRNAs of hsa_circ_0009035 were searched by the prediction programs Circinteractome (https://circinteractome.nia.nih.gov/) and Starbase (http://starbase.sysu.edu.cn/). The target genes of miR-889-3p were predicted using the online algorithms Starbase and Targetscan (http://www.targetscan.org/vert_71/?tdsourcetag=s_pcqq_aiomsg).

### Dual-luciferase reporter assay.

The fragments of the HOXB7 3′ UTR encompassing either an intact or a mutated miR-889-3p binding sequence were synthesized by BGI and individually cloned into the pMIR-REPORT vector (Promega, Leiden, The Netherlands). HeLa and Siha cells at ∼60% confluence in 24-well plates were cotransfected with 200 ng of each reporter construct, 20 ng of pRL-TK control vector (Promega), and 50 nM miR-889-3p mimic or negative mimic control. Each sample was assayed in triplicate using the dual-luciferase assay system as per the manufacturing instructions (Promega).

### RIP and RNA pulldown assays.

HeLa and Siha cells were homogenized in xTractor buffer as per the manufacturer’s protocol. In RIP assays, cell lysates were incubated with protein A/G beads (Invitrogen) conjugated with Argonaute2 antibody (AGO2; MA5-23515; Invitrogen) or isotype IgG control (10400C; Invitrogen) overnight at 4°C. In RNA pulldown assays, cell lysates were incubated with a biotin-labeled miR-899-3p mimic (Bio-miR-889-3p; Ribobio), a biotinylated antisense oligomer (CTTTAGTCT) spanning the junction of hsa_circ_0009035 (Bio-anti-hsa_circ_0009035), or a negative control (Bio-NC; Ribobio) for 3 h at 4°C before addition of the streptavidin beads (Thermo Fisher Scientific) for 3 h. In both assays, total RNA was prepared from the beads for the assessment of hsa_circ_0009035, HOXB7, and miRNA levels.

### Animal studies.

All animal procedures and experimental protocols were approved by the Animal Care and Use Committee of The First Affiliated Hospital of University of South China. Approximately 5 × 10^6^ sh-NC-infected, sh-hsa_circ_0009035-transduced, lenti-NC-transduced, or lenti-hsa_circ_0009035-infected HeLa cells were subcutaneously injected into the left flanks of female BALB/c nude mice aged 6 weeks (6 per group; Vital River Laboratory, Beijing, China). Tumor length and width were gauged every 5 days, and tumor volume was determined with the formula length × width^2^ × 0.5. All mice were euthanized after 25 days, and the xenograft tumors were collected for weight and gene expression analysis.

### Statistical analysis.

Unless otherwise indicated, means and standard deviations are representative of the average of at least three independent experiments. Statistical significance (a *P* value less than 0.05 was considered significant) was determined by a two-sided Student's *t* test, Mann-Whitney *U* test, and analysis of variance (ANOVA) with Dunnett’s multiple-comparison test. For survival data, a log-rank test was used. Correlations among hsa_circ_0009035, miR-889-3p, and HOXB7 expression levels in CC tissues were evaluated using the Spearman rank correlation test.
